# Real‐World Effectiveness and Safety of Ramipril/Indapamide Combination Therapy in Essential Hypertension: A Retrospective Study on UK Primary Care Records

**DOI:** 10.1155/ijhy/1999424

**Published:** 2026-07-13

**Authors:** Mariusz Mogielnicki, Marian Płaszczyca, Andrew Leary, Mateusz Piechaczek, Quirino Schefer, Volodymyr Stus

**Affiliations:** ^1^ Clinical Study Excellence Team, Medical Department, Zakłady Farmaceutyczne Polpharma S.A., Gdańsk, Poland; ^2^ BioStat Sp. z o.o., Rybnik, Poland; ^3^ regenold GmbH, Badenweiler, Germany

**Keywords:** adherence, drug combinations, effectiveness, hypertension, indapamide, persistence, ramipril, real-world evidence, retrospective studies, safety

## Abstract

**Objective:**

This study aimed to evaluate the patient characteristics, effectiveness, safety, adherence, and persistence of ramipril/indapamide (R/I) free combination therapy in patients with essential hypertension inadequately controlled with monotherapy.

**Methods:**

This retrospective observational cohort study used anonymized UK primary care electronic health records from IQVIA’s Medical Research Database (IMRD), incorporating “The Health Improvement Network” (THIN), a Cegedim Database. Adults with essential hypertension who switched from ramipril or indapamide monotherapy to R/I between database inception and January 1, 2021, were enrolled. Outcomes included changes in systolic blood pressure (SBP) and diastolic blood pressure (DBP), proportions achieving BP < 140/90 mmHg or SBP ≥ 20 mmHg/DBP ≥ 10 mmHg decrease, proportion of days covered (PDC), persistence, and adverse events (AEs) over 12 months.

**Results:**

A total of 1089 patients met inclusion criteria (mean age 63.5 years; 55.6% female). Mean baseline BP on monotherapy was 156.0/89.1 mmHg. Over 12 months, mean SBP decreased by 18.7 mmHg and DBP by 8.9 mmHg (both *p* < 0.001); 54.3% of patients achieved BP < 140/90 mmHg, and 61.6% met the predefined BP decrease threshold. A dose–response relationship was observed for BP outcomes. Mean PDC was 92.4% at 3 months and 86.2% at 12 months; persistence was 87.7% and 81.5%, respectively. The most frequent AE was cough (10.38%); gout occurred in 0.73%, exclusively in indapamide‐naïve patients. The outcomes remained consistent across sensitivity and subgroup analyses.

**Conclusion:**

Switching from ramipril or indapamide monotherapy to their free combination was associated with clinically meaningful BP decreases, high adherence and persistence, and a favorable safety profile in routine practice. These findings are consistent with current therapeutic guidelines that endorse its use in essential hypertension management.

## 1. Introduction

Hypertension is a major contributor to cardiovascular morbidity and mortality. Antihypertensive treatment typically combines agents from the major pharmacological groups, including blockers of the renin–angiotensin system (RAS, incorporating ACE inhibitors and sartans), calcium channel blockers (CCB), thiazide/thiazide‐like diuretics (TD), and beta‐blockers (BB) [1, 2]. The ACEI/TD combination has been recommended since the first European hypertension guidelines [3] and was elevated to first‐line treatment in the 2018 ESC/ESH Guidelines, preferably in a single‐pill combination (SPC) [4].

Ramipril and indapamide are well‐recognized drugs within these pharmacological classes. Ramipril, a widely studied ACEI, gained prominence following the landmark HOPE study published in 2000, which demonstrated its efficacy in reducing the risk of death and cardiovascular events in hypertensive patients, including those concurrently using diuretics [5]. Likewise, indapamide, a thiazide‐like diuretic, is distinguished by its more potent and long‐acting effect [1], metabolic neutrality [6], and robust evidence base supporting its cardiovascular benefits [4].

Ramipril and indapamide have been available in Europe for decades [7, 8], while the first ramipril/indapamide (R/I) SPC in the European Union was introduced in 2025 by Polpharma [9]. However, evidence on the effectiveness and safety of this combination in large, diverse populations remains limited, despite its widespread use. Building on the existing body of evidence, this study aimed to provide real‐world insights into the use and outcomes of ramipril and indapamide coadministration in essential hypertensive patients with inadequately controlled blood pressure (BP) on monotherapy.

## 2. Materials and Methods

### 2.1. Study Design

**FIGURE 1:**
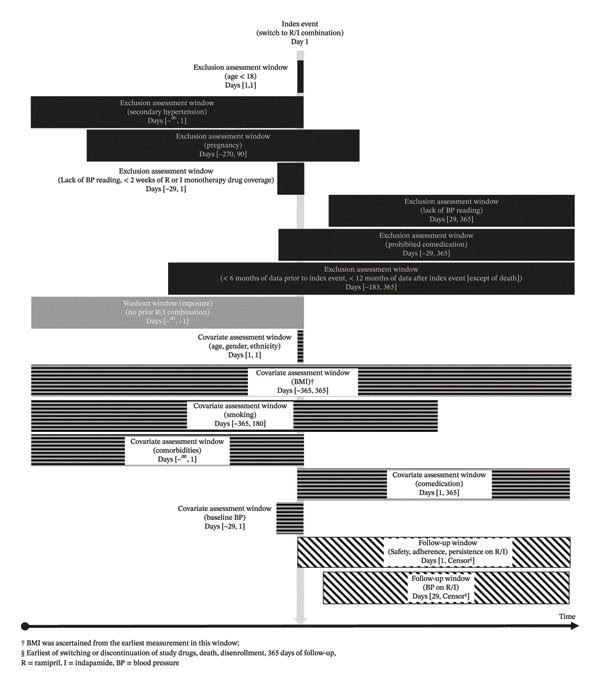
Graphical depiction of study design created based on a Creative Commons template from Schneeweiss et al. [11].

This retrospective observational cohort study used anonymized electronic health records (EHRs) to evaluate the effectiveness, safety, adherence, and persistence of the free combination of R/I in adults with essential hypertension previously treated with monotherapy with either agent. The study design is summarized in Figure 1. The STrengthening the Reporting of OBservational studies in Epidemiology guidelines (STROBE) were followed for drafting this manuscript [10].

### 2.2. Data Source

Patient‐level data were obtained from IQVIA’s Medical Research Database (IMRD), incorporating “The Health Improvement Network” (THIN), a Cegedim Database of EHRs from UK primary care practices (general practitioners [GPs]). The THIN database was selected due to its extensive coverage of approximately 5% of the UK population and its inclusion of clinically relevant information such as BP measurements, diagnoses, and prescriptions recorded during routine patient visits. This database has been widely used in numerous retrospective studies, including those on hypertension treatment [12–14].

### 2.3. Study Population

The study included adults with a diagnosis of essential hypertension (identified using READ codes), who switched from monotherapy with ramipril or indapamide to their free combination therapy and were registered in the database from its creation until January 1, 2021. Indapamide exposure in this study included both the immediate‐release 2.5‐mg formulation and the sustained‐release 1.5‐mg formulation, reflecting routine UK prescribing practice. To ensure data completeness and reliability, patients were required to have been registered with a participating practice for at least 6 months prior to and for 12 months after the index event (i.e., the date of the treatment switch). An incident user design was employed, excluding prevalent users (patients already receiving the R/I combination) to reduce selection and survivorship biases. Additional key selection criteria required patients to have at least 1 BP reading until the index event and 1 BP reading after the index event to enable the assessment of treatment effectiveness. Patients receiving other antihypertensives were excluded to isolate the effects of the R/I combination. Follow‐up was limited to the first 12 months after the index event.

### 2.4. Study Outcomes

The study evaluated five domains: patient characteristics, treatment effectiveness, adherence, persistence, and safety. Patient characteristics included demographic variables such as age, sex, ethnicity, and smoking status, as well as clinical parameters like body mass index (BMI), baseline BP classification, selected comorbidities, and concomitant medications. Effectiveness was assessed by examining changes in systolic and diastolic BP from baseline to follow‐up, the proportion of patients achieving target BP values (< 140/90 mmHg), and the proportion achieving a clinically significant decrease in BP (i.e., systolic blood pressure [SBP] ≥ 20 mmHg and/or diastolic blood pressure [DBP] ≥ 10 mmHg). BP values were derived from routine primary care recordings available in the database. Safety outcomes included the incidence of events of interest, identified using READ codes and coded according to MedDRA Version 24.0. These outcomes were expressed as the number and proportion of patients experiencing a given adverse event (AE), the number and proportion of recorded instances of a given AE relative to the total number of recorded events, and the AE rate (number per 100 patient‐years). Additionally, major adverse cardiovascular events (MACE), including cardiovascular death, stroke, and myocardial infarction, were assessed, along with overall mortality. Treatment adherence was measured as the proportion of days covered (PDC), while treatment persistence was defined as the proportion of patients continuing R/I combination therapy. The study outcomes were assessed at 3 months and 12 months after the index event.

### 2.5. Statistical Analysis

Statistical analyses were conducted using R Version 4.0.3 (R Core Team, 2020). Descriptive statistics were used to summarize patient characteristics and study outcomes. Quantitative variables were described using counts, means, standard deviations, medians, interquartile ranges, and ranges, while categorical variables were presented as numbers and percentages. Paired Student’s *t*‐tests were employed to compare the BP values before and after the switch. Multivariable linear regression models and logistic models were used to assess associations between patient characteristics and study outcomes, with a backward stepwise procedure based on Akaike information criteria (AIC) used to select optimal models. Then, the models were verified by visual analysis of plots of residuals and a goodness‐of‐fit test (*R* ^2^ for linear and Hosmer–Lemeshow test for logistic models). Statistical significance was set at *p* < 0.05, and 95% confidence intervals were calculated for all key estimates. Subgroup analyses were performed to explore variations in outcomes based on predefined patient characteristics. Sensitivity analyses were conducted to test the robustness of the findings by relaxing the selection criteria regarding data availability and timing of BP measurements.

### 2.6. Study Approval and Consent

The study protocol was reviewed and approved by the Scientific Review Committee (SRC, SRC number 21SRC018). As the IMRD database comprises anonymized electronic health records, patient informed consent was not required.

## 3. Results

### 3.1. Flowchart of Patients

**FIGURE 2:**
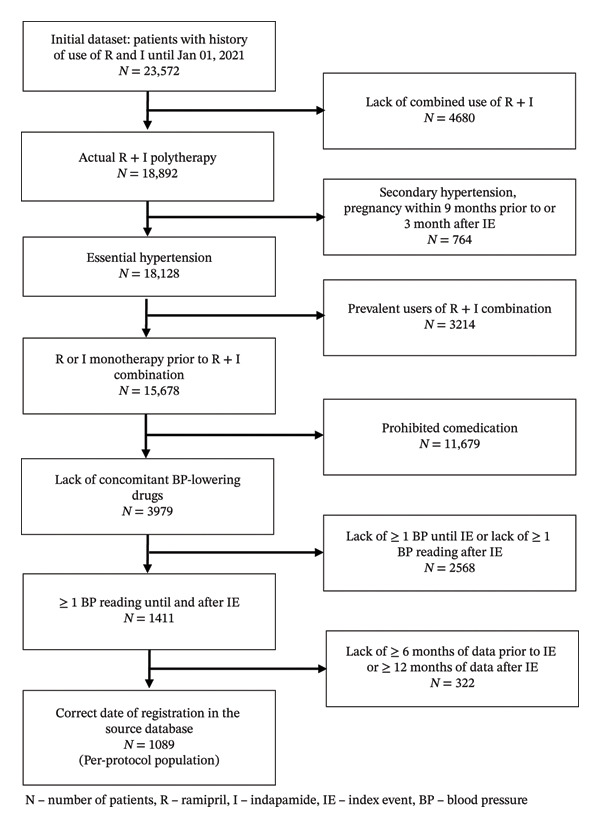
Patient disposition flowchart.

A total of 23,572 patients in the database had a history of ever receiving ramipril and indapamide until January 1, 2021. Among these, 18,892 were identified as having received the two drugs as an actual polytherapy regimen. Of this group, 18,128 patients were confirmed to have a diagnosis of essential hypertension. After applying the rest of the study’s selection criteria, 1089 patients were included in the primary analysis. The detailed disposition of patients is illustrated in Figure 2.

Sensitivity analyses with relaxed patient selection criteria included a reduced required registration period in the database (at least 1 month prior to and 2 months following the index event) and an extended allowable time window for postindex BP measurements (up to 548 days). These adjustments increased the sample size only modestly, reaching a maximum of 1333 patients.

### 3.2. Patient Characteristics

Table 1 summarizes demographic and clinical characteristics of the study population. While some demographic and lifestyle data were incomplete (notably ethnicity), key trends were observed. The mean age of the patients was 63.5 years (range: 26–93), with females slightly outnumbering males (55.6%). Among patients with available ethnicity data, the majority were White. Most patients (79%) had baseline BP corresponding to Grade 1 or Grade 2 hypertension, and the majority of those with recorded BMI data were classified as overweight or obese (83.8%), with a mean BMI of 31.1 kg/m^2^ across the population. Among patients with available smoking status data, 14.2% were current smokers. Additionally, 20% of patients had a diagnosis of diabetes, and 43.8% were on concomitant statin therapy.

**TABLE 1:** Characteristics of patients included in the per‐protocol analysis.

Patients’ characteristics	Value	*N* = 1089	%
Sex	Female	606	55.6
	Male	483	44.4

Age (years)	Mean (SD)	63.5 (11.9)	n/a
	Median	63.0	n/a
	Q1, Q3	55.0, 72.0	n/a

BMI	Mean (SD)	31.1 (6.3)	n/a
	Median	30.3	n/a
	Q1, Q3	26.6, 34.6	n/a

BMI classification	Normal	140	12.9
	Overweight	270	24.8
	Obese	453	41.6
	Missing	226	20.8

Ethnic group	African	8	0.7
	Asian	17	1.6
	Chinese	2	0.2
	Other	7	0.6
	White	459	42.1
	Missing	596	54.7

Current smoker	Yes	130	11.9
	No	787	72.3
	Missing	172	15.8

Baseline BP classification	Normal	33	3.0
	High normal (SBP 130–139 and/or DBP 85–89)	75	6.9
	Grade 1 hypertension (SBP 140–159 and/or DBP 90–99)	444	40.8
	Grade 2 hypertension (SBP 160–179 and/or DBP 100–109)	416	38.2
	Grade 3 hypertension (SBP ≥ 180 and/or DBP ≥ 110)	121	11.1
	Isolated systolic hypertension (SBP ≥ 140 and DBP < 90)^∗^	425	39.0

Monotherapy drug	Ramipril	828	76.0
	Indapamide	261	24.0

Comorbidities	Diabetes mellitus (incl subtypes)	218	20.0
	Ischemic coronary artery disorders	41	3.8

Concomitant medication	Statin	477	43.8
	Metformin	124	11.4
	Low‐dose aspirin	125	11.5

*Note:* N, number of patients; Q1–Q3, first and third quartiles.

Abbreviations: BMI, body mass index, BP, blood pressure; DBP, diastolic blood pressure; n/a, not applicable; SBP, systolic blood pressure; SD, standard deviation.

^∗^Out of 1089 subjects included in the analysis.

During the monotherapy, 76.0% of patients were treated with ramipril, with the most common daily dose being 10 mg (59.9%), followed by 5 mg (18.6%) and 2.5 mg (17.0%). A small proportion of patients used 1.25 mg, and isolated cases were reported at 7.5 mg. For patients on indapamide monotherapy, the most frequently used dose was 2.5 mg daily (69.4%), with the remaining patients using 1.5 mg.

During the combination therapy period, the most common dose of ramipril was 10 mg (52.0%), while 2.5 mg was the predominant dose of indapamide (70.7%). The most frequently prescribed daily combination was 10 mg of ramipril with 2.5 mg of indapamide (37.3%), followed by 2.5 mg + 2.5 mg (17.4%), 10 mg + 1.5 mg (14.7%), and 5 mg + 2.5 mg (10.2%). Other dose combinations were used less frequently (data not shown).

### 3.3. Effectiveness

Over the 12 months of observation, the mean SBP decreased from an average of 156.0 mmHg during monotherapy to 137.3 mmHg during combination therapy, representing a decrease of 18.7 ± 16.8 mmHg (*p* < 0.001). Similarly, DBP showed a decrease of 8.9 ± 9.8 mmHg (*p* < 0.001), from an average of 89.1 mmHg to 80.1 mmHg (Table 2).

**TABLE 2:** Evolution of blood pressure.

Follow‐up interval	Treatment phase	N	SBP (mmHg)		DBP (mmHg)	
			Mean ± SD	*p* value	Mean ± SD	*p* value
Three‐month timeframe	Baseline monotherapy	738	156.6 ± 16.2	< 0.001	89.2 ± 11.1	< 0.001
	Combination therapy	738	138.5 ± 13.4		80.5 ± 9.1	
	Difference	738	−18.1 ± 17.0		−8.7 ± 10.1	

Twelve‐month timeframe	Baseline monotherapy	1089	156.0 ± 16.6	< 0.001	89.1 ± 11.2	< 0.001
	Combination therapy	1089	137.3 ± 12.1		80.1 ± 8.2	
	Difference	1089	−18.7 ± 16.8		−8.9 ± 9.8	

*Note:* *p* values for a difference from baseline values were calculated using a two‐sided paired Student’s *t*‐test. N, number of patients, statistical significance was set at *p* < 0.05.

Abbreviations: DBP, diastolic blood pressure; SBP, systolic blood pressure; SD, standard deviation.

The addition of indapamide to ramipril was associated with a slightly greater decrease in SBP and DBP compared to the addition of ramipril to indapamide (by 19.4/9.0 mmHg vs. 16.7/8.5 mmHg, respectively, over the 12‐month period).

A subgroup of patients who adjusted the dose of either ramipril or indapamide during combination therapy while maintaining a constant dose of the other drug experienced greater BP decreases. The analysis compared BP values between higher and lower doses of each drug, irrespective of whether the dose was increased or decreased. Specifically, patients receiving a higher dose of ramipril experienced an additional decrease in SBP and DBP of 11.1 mmHg and 4.8 mmHg, respectively, while those receiving a higher dose of indapamide experienced additional decreasesof 3.1 mmHg in SBP and 2.1 mmHg in DBP.

During the 12 months of combination therapy, 54.3% (95% CI: 51.2–57.4) of patients achieved BP target values (< 140/90 mmHg), while 61.6% (95% CI: 58.6–65.5) achieved the defined BP decrease thresholds (SBP ≥ 20 mmHg and/or DBP ≥ 10 mmHg).

The BP‐related outcomes remained consistent across various analytical approaches, including assessments over a 3‐month timeframe (Table 2), analyses of the last BP measurements before and after the index event, and evaluations using stricter or more liberal selection criteria (Table 3). For example, in a subset of the per‐protocol population with at least one BP reading within 3 months after the index event, SBP decreased from an average of 156.6 mmHg during monotherapy to 138.5 mmHg on combination therapy, a decrease of 18.1 ± 17.0 mmHg (*p* < 0.001). Similarly, DBP decreased by 8.7 ± 10.1 mmHg (*p* < 0.001), from 89.2 mmHg to 80.5 mmHg, during the same period.

**TABLE 3:** Evolution of blood pressure over 12 months across different analytical approaches.

Analysis category	Analysis description	*N*	Mean change in SBP ± SD (mmHg)	Mean change in DBP ± SD (mmHg)
Primary	Main analysis of the per‐protocol dataset	1089	−18.7 ± 16.8	−8.9 ± 9.8

Secondary	Last available BP measurement before and after the index event	1089	−20.8 ± 19.1	−9.9 ± 11.9
	Subset of patients with at least 3 BP measurements before and after the index event	79	−18.6 ± 14.1	−8.3 ± 8.4

Sensitivity	Expanded population with reduced registration‐time requirements (≥ 1 month before and ≥ 2 months after the index event)	1256	−18.6 ± 16.9	−8.9 ± 9.8

*Note:* N, number of patients.

Abbreviations: DBP, diastolic blood pressure; SBP, systolic blood pressure; SD, standard deviation.

Regression analyses linked greater BP decreases with higher baseline BP, indapamide as an add‐on drug, higher doses of ramipril and indapamide, 100% adherence, and increasing age (but only for DBP; for SBP, the association was the opposite, Supporting 1).

### 3.4. Adherence

Mean PDC was 92.4% (median = 100.0%, IQR: 96.7%–100.0%) during the first 3 months, which decreased to 86.2% (median = 99.7%, IQR: 89.0%–100.0%) over the full 12‐month observation period. Prescription data indicated that 67.3% of patients had complete medication coverage (i.e., PDC = 100.0%) during the initial 3 months, while this proportion declined to 47.8% by the end of the 12‐month period.

Findings were consistent across subgroups and sensitivity analyses. Regression analyses showed that the main predictors of full treatment adherence were older age and White ethnicity (Supporting 1).

### 3.5. Persistence

Among the per‐protocol population, 955 patients (87.7%, 95% CI: 85.6–89.6) remained on combination therapy for more than 3 months. Persistence showed a slight decline over time, with 81.5% of patients (95% CI: 79.1–83.8) continuing the therapy through the full 12‐month follow‐up period.

### 3.6. Safety

A total of 185 AEs were reported during the 12 months following the index event. The most frequently reported event was cough, with 158 occurrences (85.41% of the recorded number of events) affecting 113 patients (10.38% of the per‐protocol population). Gout was the second most common event, with 10 occurrences affecting 8 patients (0.73% of the per‐protocol population). Orthostatic hypotension was reported in 4 patients, and impaired glucose tolerance was reported in 4 patients (Table 4).

**TABLE 4:** Summary of adverse events of interest in the per‐protocol population during the 12 months of follow‐up.

SOC name	PT name	Number and proportion of recorded instances of given AE relative to the total number of recorded events (*N* = 185)	Number and proportion of patients experiencing given AE (*N* = 1089)	Adverse event rate (number/100 patient‐years)
Cardiac disorders	Acute myocardial infarction	2 (1.08%)	1 (0.09%)	0.184

Investigations	Blood cholesterol increased	1 (0.54%)	1 (0.09%)	0.092
	Blood electrolytes abnormal	1 (0.54%)	1 (0.09%)	0.092
	Blood glucose abnormal	1 (0.54%)	1 (0.09%)	0.092

Metabolism and nutrition disorders	Glucose tolerance impaired	4 (2.16%)	4 (0.37%)	0.368
	Gout	10 (5.41%)	8 (0.73%)	0.919
	Hyperuricemia	1 (0.54%)	1 (0.09%)	0.092

Respiratory, thoracic, and mediastinal disorders	Cough	158 (85.41%)	113 (10.38%)	14.519
	Hemoptysis	1 (0.54%)	1 (0.09%)	0.092

Skin and subcutaneous tissue disorders	Urticaria	2 (1.08%)	2 (0.18%)	0.184

Vascular disorders	Orthostatic hypotension	4 (2.16%)	4 (0.37%)	0.368

*Note:* N, number of patients; coded using MedDRA dictionary Version 24.0.

Abbreviations: PT, Preferred term; SOC, System Organ Class.

One patient experienced a MACE, specifically two episodes of myocardial infarction. Additionally, three patients died within the 12‐month follow‐up period. Of these, one death was attributed to glioblastoma multiforme, while the causes of death for the other two patients were not recorded.

The results remained consistent across subgroup and sensitivity analyses, except for gout, which occurred only in patients naïve to indapamide.

## 4. Discussion

This study enrolled a large and heterogeneous population ranging widely in age, BMI, and smoking status. However, it is important to note that a substantial proportion of the data was missing for selected variables, which may limit the generalizability of our findings, particularly to more ethnically diverse populations. Incomplete ethnicity recording is a recognized challenge in retrospective studies utilizing routine healthcare databases [13]. Nonetheless, the characteristics of the study population correspond to those reported in other large retrospective studies on British hypertensive patients [13, 14]. Additionally, the characteristics align with studies on Polish [15] and Spanish hypertensive patients [16], supporting its representativeness in a broader context. The higher number of patients treated initially with ramipril than indapamide monotherapy (828 vs. 261) reflects prescribing practices in the United Kingdom, where ACEIs were recommended as first‐line therapy for uncomplicated essential hypertension, in line with British hypertension treatment guidelines of the time [17, 18].

Mean baseline BP on monotherapy (156.6/89.2 mmHg) aligns with expectations for patients who had not responded adequately to monotherapy [15, 19, 20]. This lack of response prompted physicians to initiate a second agent, making these patients eligible for the study. The observed falls in SBP and DBP of 18.7 ± 16.8 mmHg and 8.9 ± 9.8 mmHg are clinically significant [21, 22] and are consistent with clinical experience supporting the superiority of dual combination over monotherapy [19, 20]. However, regression to the mean and time‐related effects associated with treatment escalation represent plausible alternative explanations for part of the observed BP decrease. Nevertheless, the observed SBP/DBP decrease is generally within the range of effect sizes reported in other studies evaluating similar combinations. For example, randomized controlled trials (RCTs) with an add‐on design reported SBP/DBP reductions of 11.6/10.6 mmHg for ramipril/hydrochlorothiazide [23], 11.4/7.4 mmHg for candesartan/hydrochlorothiazide [24], 10.9–11.7/8.4–8.9 mmHg for moexipril/hydrochlorothiazide [25], and 6.0/7.4 mmHg for lisinopril/hydrochlorothiazide [26]. In comparison, a more pronounced decrease of 30.0/14.8 mmHg was reported in another study of R/I (KONSONANS) [27]. The variability in BP falls across studies can be attributed to differences in study design and population characteristics [28]. For instance, the larger BP decreases observed in KONSONANS may be explained by the inclusion of a subset of treatment‐naïve patients (18.3%); moreover, the study treatment was an SPC, which is known to enhance adherence and potentially improve treatment outcomes [1, 2, 27, 29]. Importantly, we observed a clinically meaningful decrease in BP despite the inclusion of a broad patient population comprising a substantial proportion of obese individuals, a group commonly associated with more difficult‐to‐control or resistant hypertension [1, 15].

At baseline, patients on ramipril monotherapy had very similar BP values to those on indapamide monotherapy (156.1/89.2 mmHg and 155.8/88.7 mmHg, respectively); however, the BP decrease was larger for patients who had indapamide added to ramipril (19.4/9.0 mmHg) than when ramipril was added to indapamide (16.7/8.5 mmHg). This difference may be linked to the dosing recommendations outlined in the SmPCs of the respective monoproducts [30–32]. Specifically, the majority of patients taking ramipril as the initial agent were uptitrated to the maximum dose (10 mg) before indapamide add‐on was introduced, which was coprescribed at its maximum (and only) dose. In contrast, while patients taking indapamide as the initial agent were also on the maximum dose (2.5 mg immediate release or 1.5 mg sustained release), ramipril as the second agent was in many cases added in line with its SmPC at a comparatively low dose of 2.5 mg, with some patients receiving as little as 1.25 mg. Considering the dose‐dependent BP‐lowering efficacy of ramipril [33], this dosing pattern likely contributed to the slightly smaller BP decrease when ramipril was added to indapamide.

In a subgroup of patients who adjusted the dose of either ramipril or indapamide (while maintaining an unchanged dose of the second drug), additional decreases in SBP and DBP were observed using a higher dose of ramipril (−11.1 mmHg and −4.8 mmHg, respectively) or indapamide (−3.1 mmHg and −2.1 mmHg, respectively). This aligns with the known dose–response relationship for ramipril [33], whereas the less pronounced association with higher doses of indapamide can be attributed to the limited BP‐lowering benefit of escalating its dose [31, 32]. Nevertheless, it may also suggest that the immediate‐release formulation of indapamide (2.5 mg) may be slightly more effective in combination with ramipril than the sustained‐release formulation (1.5 mg). This corresponds with the general principle that combining two drugs with complementary mechanisms of action produces a steeper dose–response effect [1, 2]. However, this should be interpreted cautiously due to the small sample size of this subset and conflicting evidence regarding the relative efficacy of the two indapamide formulations. Although some numerical differences favoring the 2.5 mg IR formulation have been reported, such as a 2.3/0.8 mmHg advantage in 24‐h ABPM, most studies have concluded their equivalency [34–36].

The proportion of patients achieving treatment response, i.e., 54.3% of patients with BP < 140/90 mmHg and 61.6% with a decrease of SBP ≥ 20 mmHg and/or DBP ≥ 10 mmHg, is in agreement with the general observation that about half to two‐thirds of patients achieve BP control with dual therapies [1, 37]. However, this was lower than the response rate reported in the KONSONANS study, where 98.8% of patients achieved BP < 140/90 mmHg after 6 months of therapy [28]. As previously discussed, this cross‐study difference can be attributed to differences in study design.

The mean adherence value was high (86.2% of the 12 months of observation) and exceeded the 80% threshold, which reflects the current consensus for sufficient adherence when expressed as PDC [38, 39]. Furthermore, it is comparable to the highest rates reported in other studies on antihypertensive treatments, where nonadherence rates ranged from 9% to 47% [40]. However, it should be noted that adherence estimated using PDC reflects medication availability rather than confirmed ingestion [38].

The treatment persistence was above 80% after 12 months of combination therapy, aligning with the highest 1‐year rates reported in large retrospective studies on hypertensive patients. For comparison, persistence rates have been reported as 43% in Lithuanian [41], 59.5%–65.2% in Korean [42], 74% in Swedish [43], and 78%–91% in Canadian patients [44]. However, the persistence observed in this study was lower than the 97.5% reported in the KONSONANS study evaluating the same combination over a shorter 6‐month period [27]. This difference may be attributed to the use of an SPC in KONSONANS, which is known to enhance persistence [1, 2] and its prospective design, where patients’ awareness of observation may have influenced their behavior [45].

The relatively high values found for adherence and persistence are in keeping with the nature of the health service in the United Kingdom, where patients with chronic conditions such as essential hypertension benefit from continuity of care provided by a designated GP practice. This system ensures regular follow‐up and care that is free at the point of service, while prescriptions are also provided free of charge, incentivizing patients to have them filled [46–48]. In addition, the relatively favorable safety and tolerability of the study treatment (discussed in the next section) may also have contributed to the observed adherence and persistence rates.

The most common AE detected in patient records in the period after the index date was cough, a well‐documented AE associated with ACEI [1, 2, 30], reported in 10.38% of patients. Gout occurred in 8 patients, while other events were reported in isolated cases, such as impaired glucose tolerance or orthostatic hypotension, each seen in 4 patients. Gout was observed exclusively in indapamide‐naïve patients, which aligns with the observation that diuretics are a major cause of secondary hyperuricemia and gout [49]. While this remains a crude assessment of the safety of combination therapy, the nature and incidence of AEs observed are within the ranges described in the SmPCs for marketed products containing indapamide and ramipril [30–32]. These findings also align with the broader conclusion that ACEI/TD combinations have a favorable safety profile [50]. The number of reported AEs in this study is higher than the number reported in the KONSONANS study, which documented 14 AEs, including 5 cases of headache and 3 cases of cough [27], likely reflecting differences in patient characteristics (e.g., age and BMI distribution), the larger sample size (1089 vs. 524 patients), and the longer observation period (12 months vs. 6 months), thereby enabling detection of more AEs in our study. This corresponds to the current perception of the value of retrospective real‐world studies in providing a comprehensive assessment of safety, particularly over extended periods [45]. However, it should be noted that AEs in this study were identified from READ‐coded records from routine primary care rather than through systematic AE collection, including direct review of raw laboratory data, which may have resulted in under‐ascertainment of some events, including those managed outside the primary care setting.

The sensitivity analyses conducted on expanded populations and subgroup analyses yielded results consistent with those observed in the per‐protocol population. Findings remained stable across both stricter and more relaxed selection criteria. Regression analyses identified factors associated with improved BP‐related outcomes and adherence, which aligned with existing knowledge about hypertensive patients and their treatment. These concordant results reinforce the generalizability of the findings.

This study, to the best of the authors’ knowledge, represents the largest investigation into the clinical performance of the R/I combination, although it has several limitations inherent to its single‐arm, noncontrolled, and retrospective design, including the absence of an active comparator, which precludes definitive causal inference. The results of retrospective studies are generally regarded as providing lower‐level evidence compared to standard prospective RCTs, including hypertension research [1, 2]. This is due, inter alia, to the potential lack of complete data, including missing demographic and lifestyle variables, as well as insufficient endpoint data (e.g., BP readings at standardized time points). However, perceptions of the relative value of RCTs and the approach used in this retrospective study have evolved, with recognition that both approaches have their strengths and limitations. Indeed, RCTs remain the “gold standard” in clinical research, but their rigid selection criteria and predefined procedures create a “hermetic” environment that often excludes parts of the targeted population and may not reflect routine clinical practice. In contrast, real‐world studies involve large, heterogeneous populations that have been subjected to actual clinical practice in all its variety, providing a broader and more practical perspective on treatment outcomes. A limitation inherent to the retrospective design can also be considered a strength of the study, due to the lack of awareness of observation, which is a known factor affecting patient and investigator behavior during a prospective trial [1, 45].

## 5. Conclusions

This study provides evidence supporting clinically relevant decreases in BP following the addition of indapamide to ramipril, or vice versa, along with favorable safety, adherence, and persistence profiles. While acknowledging the methodological constraints of its design, this study offers valuable real‐world insights into the clinical performance of the ramipril and indapamide combination. Given the inclusion of a large, heterogeneous population of hypertensive patients, the findings may be generalizable to hypertensive populations with similar demographic and clinical profiles in comparable healthcare settings.

## Funding

This study was entirely funded by Zakłady Farmaceutyczne Polpharma S.A.

## Disclosure

The authors declare that the interests outlinedin the “Conflicts of Interest” section did not influence the design, conduct, analysis, interpretation, or reporting of the study and that the results and conclusions presented are an accurate and unbiased reflection of the data.

## Conflicts of Interest

Mariusz Mogielnicki and Volodymyr Stus are employees of Zakłady Farmaceutyczne Polpharma S.A., which sponsored the study and may have an interest in the outcomes. Marian Płaszczyca and Mateusz Piechaczek are employees of Biostat Sp. z o.o., and Andrew Leary and Quirino Schefer are employees of regenold GmbH; both organizations were contracted by Polpharma for study planning and conduct.

## Supporting Information

Additional supporting information can be found online in the Supporting Information section.

## Supporting information


**Supporting Information** Supporting 1 provides a summary of the multivariable regression analyses performed in this study, including model specifications, candidate covariates, the variable‐selection approach, and effect estimates for selected blood pressure and adherence outcomes.

## Data Availability

The data that support the findings of this study are available from the corresponding author upon reasonable request.
