# Supplementary material for: Real‐World Effectiveness and Safety of Ramipril/Indapamide Combination Therapy in Essential Hypertension: A Retrospective Study on UK Primary Care Records
Source: Int J Hypertens. 2026 Jul 13;2026:1999424. doi: 10.1155/ijhy/1999424 (PMC13365794; doi:10.1155/ijhy/1999424)
Supplement: Supplementary file 1 — Supporting Information Supporting 1 provides a summary of the multivariable regression analyses performed in this study, including model specifications, candidate covariates, the variable‐selection approach, and effect estimates for selected blood pressure and adherence outcomes. [file IJHY-2026-1999424-s001.docx]

# **Supplement 1**

# **Summary of Regression Analyses**

This document provides an overview of the regression model specifications utilized within the study and presents a succinct tabular summary of candidate covariates and associated effect estimates for selected endpoints.

Candidate covariates were determined a priori for each model. Final models were developed via backward stepwise selection guided by the Akaike Information Criterion (AIC); consequently, tables display coefficients or odds ratios exclusively for covariates retained after this selection process. Missing values were not imputed; observations lacking covariate data were excluded, resulting in certain models being fitted on subsets with complete information.

Separate summary tables are included below for each selected study outcome.

Legend:

- “+” indicates inclusion of the covariate as a candidate in the initial model specification;
- “−” denotes exclusion of the covariate as a candidate;
- “NR” signifies that the covariate was a candidate (“+”) but is not reported in the final output table (not retained after AIC selection and/or omitted due to presentation of only the final model);
- “NA” refers to cases where the covariate was not relevant for the particular model specification.
- Effect formats: Linear models present β (95% CI) and p-values; logistic models report OR (95% CI) and p-values.

## A. SBP difference (linear regression)

| **Covariate** | **Basic model** | | **Basic model without ethnicity** | | **Model with adherence and without ethnicity** | |
| --- | --- | --- | --- | --- | --- | --- |
|  | **Considered (+/-)** | **Effect estimate [mmHg]** | **Considered (+/-)** | **Effect estimate [mmHg]** | **Considered (+/-)** | **Effect estimate [mmHg]** |
| **Adherence: 100%** | - | NA | - | NA | + | -2.388 (-3.976 to -0.800), **p=0.003** |
| **Age** | + | 0.210 (0.094 to 0.325), **p=0.000** | + | 0.163 (0.090 to 0.236), **p=0.000** | + | 0.172 (0.098 to 0.245), **p=0.000** |
| **BMI** | + | 0.156 (-0.039 to 0.351), p=0.116 | + | 0.146 (0.014 to 0.278), **p=0.031** | + | 0.131 (-0.001 to 0.263), p=0.051 |
| **Coexisting diabetes** | + | NR | + | NR | - | NA |
| **Coexisting ischaemic coronary artery disorder** | + | NR | + | NR | - | NA |
| **Current smoking** | + | NR | + | NR | + | NR |
| **indapamide dose** | + | -2.155 (-4.955 to 0.644), p=0.131 | + | -1.830 (-3.583 to -0.078), **p=0.041** | - | NA |
| **Initial drug: ramipril** | + | NR | + | NR | + | -1.686 (-3.552 to 0.180), p=0.076 |
| **Race: non white** | + | NR | - | NA | - | NA |
| **Ramipril dose** | + | NR | + | -0.295 (-0.515 to -0.074), **p=0.009** | - | NA |
| **SBP value prior to the index event** | + | -0.749 (-0.826 to -0.673), **p=0.000** | + | -0.749 (-0.798 to -0.701), **p=0.000** | + | -0.750 (-0.799 to -0.701), **p=0.000** |
| **Sex: male** | + | NR | + | NR | + | NR |

## B. DBP difference (linear regression)

| **Covariate** | **Basic model** | | **Basic model without ethnicity** | | **Model with adherence and without ethnicity** | |
| --- | --- | --- | --- | --- | --- | --- |
|  | **Considered (+/-)** | **Effect estimate [mmHg]** | **Considered (+/-)** | **Effect estimate [mmHg]** | **Considered (+/-)** | **Effect estimate [mmHg]** |
| **Adherence: 100%** | - | NA | - | NA | + | -1.991 (-2.947 to -1.034), **p=0.000** |
| **Age** | + | -0.182 (-0.253 to -0.112), **p=0.000** | + | -0.136 (-0.184 to -0.088), **p=0.000** | + | -0.132 (-0.179 to -0.084), **p=0.000** |
| **BMI** | + | NR | + | 0.082 (0.001 to 0.162), **p=0.047** | + | 0.061 (-0.019 to 0.140), p=0.133 |
| **Coexisting diabetes** | + | NR | + | -1.101 (-2.242 to 0.040), p=0.059 | - | NA |
| **Coexisting ischaemic coronary artery disorder** | + | 2.732 (-0.956 to 6.419), p=0.146 | + | NR | - | NA |
| **Current smoking** | + | NR | + | NR | + | NR |
| **DBP value prior to the index event** | + | -0.691 (-0.763 to -0.619), **p=0.000** | + | -0.692 (-0.741 to -0.643), **p=0.000** | + | -0.684 (-0.731 to -0.636), **p=0.000** |
| **Indapamide dose** | + | -1.844 (-3.501 to -0.186), **p=0.029** | + | -1.509 (-2.569 to -0.448), **p=0.005** | - | NA |
| **Initial drug: ramipril** | + | NR | + | NR | + | NR |
| **Race: non white** | + | NR | - | NA | - | NA |
| **Ramipril dose** | + | NR | + | NR | - | NA |
| **Sex: male** | + | NR | + | NR | + | NR |

## C. Achieving SBP <140 mmHg and/or DBP <90 mmHg (logistic regression)

| **Covariate** | **Basic model** | | **Basic model without ethnicity** | | **Model with adherence and without ethnicity** | |
| --- | --- | --- | --- | --- | --- | --- |
|  | **Considered (+/-)** | **Effect estimate [OR]** | **Considered (+/-)** | **Effect estimate [OR]** | **Considered (+/-)** | **Effect estimate [OR]** |
| **Adherence: 100%** | - | NA | - | NA | + | 1.836 (1.264 to 2.667), **p=0.001** |
| **Age** | + | NR | + | 1.014 (0.996 to 1.033), p=0.120 | + | NR |
| **BMI** | + | NR | + | NR | + | NR |
| **Current smoking** | + | NR | + | NR | + | NR |
| **DBP value prior to the index event** | + | 1.038 (1.013 to 1.063), **p=0.003** | + | 1.048 (1.028 to 1.068), **p=0.000** | + | 1.043 (1.026 to 1.060), **p=0.000** |
| **Coexisting diabetes** | + | NR | + | NR | - | NA |
| **Coexisting Ischaemic coronary artery disorders** | + | NR | + | NR | - | NA |
| **indapamide dose** | + | 2.193 (1.238 to 3.885), **p=0.007** | + | 1.672 (1.140 to 2.453), **p=0.009** | - | NA |
| **Initial drug: ramipril** | + | NR | + | NR | + | NR |
| **Race: non white** | + | NR | - | NA | - | NA |
| **Ramipril dose** | + | NR | + | 1.062 (1.009 to 1.117), **p=0.021** | - | NA |
| **SBP value prior to the index event** | + | NR | + | NR | + | NR |
| **Sex: male** | + | 0.622 (0.357 to 1.086), p=0.095 | + | 0.692 (0.478 to 1.003), p=0.052 | + | 0.685 (0.475 to 0.990), **p=0.044** |

## D. Achieving decrease of SBP ≥20 mmHg and/or DBP ≥10 mmHg (logistic regression)

| **Covariate** | **Basic model** | | **Basic model without ethnicity** | | **Model with adherence and without ethnicity** | |
| --- | --- | --- | --- | --- | --- | --- |
|  | **Considered (+/-)** | **Effect estimate [OR]** | **Considered (+/-)** | **Effect estimate [OR]** | **Considered (+/-)** | **Effect estimate [OR]** |
| **Adherence: 100%** | - | NA | - | NA | + | 1.375 (0.976 to 1.94), p=0.069 |
| **Age** | + | 1.03 (1.002 to 1.06), **p=0.033** | + | 1.016 (0.997 to 1.04), p=0.100 | + | NR |
| **BMI** | + | NR | + | 0.979 (0.952 to 1.01), p=0.147 | + | 0.978 (0.952 to 1.00), p=0.114 |
| **Coexisting diabetes** | + | NR | + | 1.377 (0.919 to 2.06), p=0.121 | - | NA |
| **Coexisting Ischaemic coronary artery disorders** | + | NR | + | NR | - | NA |
| **Current smoking** | + | NR | + | NR | + | 0.669 (0.402 to 1.11), p=0.121 |
| **DBP value prior to the index event** | + | 1.08 (1.05 to 1.12), **p=0.000** | + | 1.097 (1.073 to 1.12), **p=0.000** | + | 1.085 (1.065 to 1.11), **p=0.000** |
| **Indapamide dose** | + | 1.56 (0.89 to 2.74), p=0.120 | + | NR | - | NA |
| **Initial drug: ramipril** | + | NR | + | NR | + | NR |
| **Race: non white** | + | NR | - | NA | - | NA |
| **Ramipril dose** | + | NR | + | 1.048 (0.998 to 1.10), p=0.059 | - | NA |
| **SBP value prior to index event** | + | 1.05 (1.03 to 1.08), **p=0.000** | + | 1.058 (1.043 to 1.07), **p=0.000** | + | 1.061 (1.047 to 1.08), **p=0.000** |
| **Sex: male** | + | NR | + | 0.721 (0.509 to 1.02), p=0.067 | + | 0.738 (0.522 to 1.04), p=0.086 |

## E. Adherence: achieving PDC 100% (logistic regression)

| **Covariate** | **Simple model** | | **Expanded model** | |
| --- | --- | --- | --- | --- |
|  | **Considered (+/-)** | **Effect estimate [OR]** | **Considered (+/-)** | **Effect estimate [OR]** |
| **Age** | + | 1.019 (1.007 to 1.030), **p=0.001** | + | NR |
| **BMI** | - | NA | + | NR |
| **Current smoking** | - | NA | + | NR |
| **DBP value prior to index event** | + | 1.010 (0.998 to 1.022), p=0.117 | + | 0.984 (0.965 to 1.002), p=0.085 |
| **Initial drug: ramipril** | + | NR | + | NR |
| **Race: non white** | - | NA | + | 0.304 (0.119 to 0.779), **p=0.013** |
| **SBP value prior to index event** | + | NR | + | NR |
| **Sex: male** | + | NR | + | NR |
